# Synthesis and Characterization of Nanostructured Thorium Carbide for Radioactive Ion Beam Production

**DOI:** 10.3390/nano16020127

**Published:** 2026-01-18

**Authors:** Edgar Reis, Pedro Amador Celdran, Olaf Walter, Rachel Eloirdi, Laura Lambert, Thierry Stora, Simon Stegemann, Doru C. Lupascu, Sebastian Rothe

**Affiliations:** 1 European Organization for Nuclear Research (CERN), Esplanade des Particules 1, 1211 Geneva, Switzerlandsimon.thomas.stegemann@cern.ch (S.S.);; 2 Institute for Materials Science, University of Duisburg-Essen, 45141 Essen, Germany; doru.lupascu@uni-due.de; 3 Institute for Transuranium Elements, Joint Research Center, 76125 Karlsruhe, Germany

**Keywords:** thorium carbide, nanomaterials, radioactive ion beams, ISOL

## Abstract

Thorium carbide (${\mathrm{ThC}}_{2\pm x}$) nano-structured thin disc-like pellets were produced from thoria nanoparticles (${\mathrm{ThO}}_{2}$-NP) and multi-walled carbon nanotubes (MWCNT). These composites are to be studied as a target material candidate for radioactive ion beam (RIB) production via nuclear reactions upon impact with high-energy proton beams on a stack of solid pellets. The ${\mathrm{ThO}}_{2}$-NP precursor was produced via precipitation of thorium oxalate from a thorium nitrate solution with oxalic acid and subsequent hydrothermal oxidation of the oxalate, creating the thoria nanoparticles. The ${\mathrm{ThO}}_{2}$-NP were then mixed with MWCNT in isopropyl alcohol and sonicated by two different methods to create a nanoparticle dispersion. This dispersion was then heated under medium vacuum to evaporate the solvent; the resulting powder was pressed into pellets and taken to an inert-atmosphere oven, where it was heated to 1650 °C and carbothermally reduced to ${\mathrm{ThC}}_{2\pm x}$. The resulting pellets were characterized via XRD, SEM-EDS, and Raman spectroscopy. The resulting thorium pellets exhibited, at most, trace levels of the oxide precursor. Furthermore, the nanotube structures were still present in the final product and are expected to contribute positively towards faster radioisotope release times by lowering isotope diffusion times, which is required for the efficient extraction of the shortest-lived (<1 s half-life) radioisotopes.

## 1. Introduction

In the isotope separation on-line (ISOL) method, radioactive atoms are produced inside a target material through impact with a high-energy proton beam (typically ranging from 40 MeV to 1.4 GeV), which will create new isotopes though nuclear fission, spallation, and decay reactions. These created species then need to be volatilized and extracted from the target material in order to bring them to the ion source where atoms are ionized and then extracted as ion beams by application of a large potential difference (typically 20 to 60 KeV at CERN-ISOLDE). However, many of the target isotopes suffer from factors such as poor production cross-sections, short half-lives, and slow diffusion and effusion times from the target material. This leads to the requirement for a target material that balances the high density of the main element undergoing nuclear reactions and still maintaining a high enough porosity level (ideally open), while simultaneously allowing for stable operations for multiple days or even weeks at the high temperatures required to volatilize the elements of interest [1,2].

Engineered nano- and micrometer-sized materials have been studied for ISOL application in the past, with targets made from nanometric CaO [3], pure Na zeolite [4] sub-micrometric SiC [5], Y_2_O_3_ [6], ${\mathrm{UC}}_{2\pm x}$/MWCNT [1], and others [2] having already been studied under irradiation at CERN-ISOLDE and other ISOL facilities, exhibiting faster isotope release and greater thermal stability over the operation time. Uranium carbide with MWCNT has exhibited significantly increased release efficiency compared to the microstructured UC analogue [1]. Corradetti et al. previously studied thorium carbide produced with graphite and graphene oxide precursors as a possible target material [7], showcasing an increased surface area but still containing significant oxide content after the carbothermal reduction in the thorium oxide precursor, which is undesirable as the presence of oxygen hinders the volatization of many species of interest due to oxide formation (e.g., Mg, Sc, Hf, Y) and thorium oxide is known to densify at temperatures above 1700 °C [8], posing problems for the production of Ac-based beams. Chowdhury et al. attempted to produce ${\mathrm{UC}}_{2\pm x}$ by employing electrospinning to create nanostructured fibers but similarly suffered from incomplete carbothermal reduction (circa 30% of oxide in the final product) [9]. It has been reported for TiC made with MWCNT as carbon source that the MWCNT structure can effectively provide carbon to the metal to form the carbide while ensuring the nanotube structure is still present after the production process [10], which, if verified, would create low-density barriers to slowdown sintering in the target material while allowing for significantly faster diffusion and effusion times for the produced radioisotopes. The present work investigates the synthesis and the feasibility of a thorium carbide + MWCNT ISOL target material, which would combine the advantages of a high-surface-area material with excellent thermal stability, as demonstrated for its uranium-based analogue [1].

## 2. Materials and Methods

**Figure 1:**
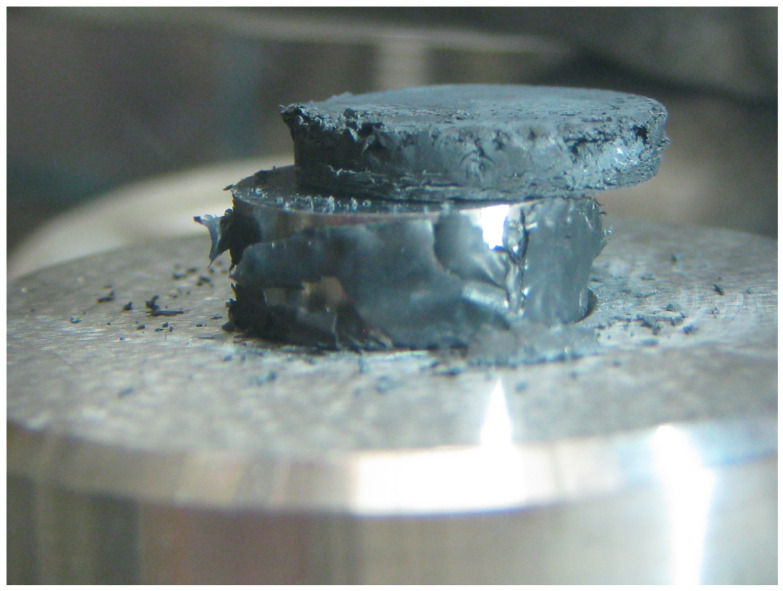
Pellet of thoria precursor with added MWCNT prior to carbothermal reduction.

The synthesis of the nanostructured thoria precursor from a thorium nitrate precursor (Merck) via oxalate route was already described in detail by O. Walter et al. [11] and will not be further mentioned herein. For the first sample (ThC-M), a dispersion of 2.65 g of nanostructured ${\mathrm{ThO}}_{2}$ powder and 35 mL of isopropyl–alcohol (IPA) was prepared inside a Schlenk flask, to which 0.8 g of MWCNT (Nanocyl NC7000) was added (carbon in excess at a 1:6 molar ratio), before the flask was placed in an ultrasonic bath for one hour to achieve a homogenous dispersion. Only a small viscosity change was observed throughout the sonic bath treatment and white thoria particle agglomerates were still visible afterwards in the suspension. For the second sample (ThC-US), a dispersion of 2.85 g of nanostructured ${\mathrm{ThO}}_{2}$ powder and 30 mL of IPA was prepared inside a Schlenk flask and 0.82 g of MWCNT was then added to this suspension. It was then mixed using an ultrasonic finger (Hielcher UP200ST) set at 40 W and 43% amplitude. In this case, a rapid and significant viscosity increase was observed within a few seconds of the start of the sonication. After 15 min of agitating, the mixture was fully homogenized. Both suspensions were then dried at circa 100 °C and the resulting powders were taken to a hydraulic pellet press (operating at 24.9 MPa), with the resulting cylindrical pellets (Figure 1) having a diameter of 15 mm and an average thickness of around 2.4 mm). Zinc stearate was employed as a lubricant on the pellet die surface to prevent pellets from sticking to the surface of the pellet die during pellet punch-out.

**Figure 2:**
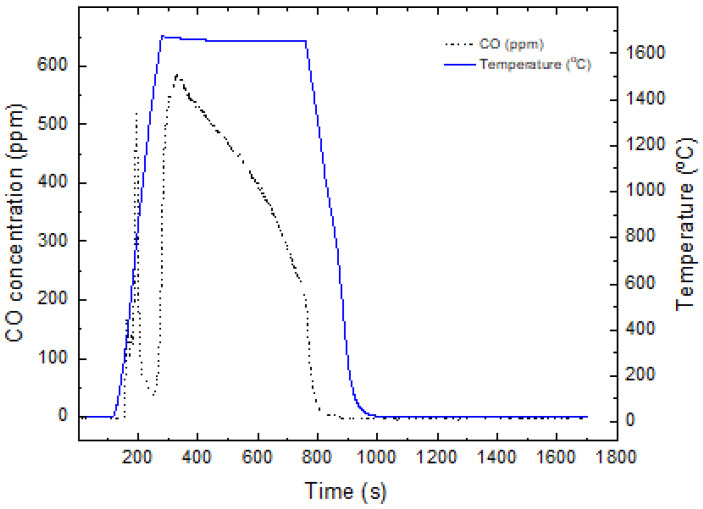
Release of carbon monoxide over time during the ${\mathrm{ThO}}_{2}$ carbothermal reduction to ${\mathrm{ThC}}_{2\pm x}$ in inert atmosphere.

The pressed ${\mathrm{ThO}}_{2}$ + MWCNT pellets from both batches were then simultaneously reduced into thorium carbide in an oven under Ar atmosphere with a temperature ramp of 10 °C/min until 1650 °C and with a dwell time of 8 h. The evolution of CO from the carbothermal reduction was monitored via a CO gas analyzer connected to the side of the exhaust of the oven (Figure 2) to follow the progress of the carbothermal reduction reaction, with a CO release peak observed after 600 °C corresponding to the oxidation of free carbon followed by the main peak, starting at 1200 to 1300 °C, indicating the start of the reduction of the thoria to thorium carbide. The kinetics of the carbothermal reduction reaction of thoria with CNT in inert atmosphere were not studied in-depth, with Kanno et al. reporting that the reaction with graphite in high-vacuum conditions is limited by the diffusion of O and C atoms through the ${\mathrm{ThC}}_{x}$ surface layer, starting at 1300 °C [12].

Samples of the converted pellets were taken for characterization via SEM-EDS, XRD, and Raman spectroscopy. Due to the expected high reactivity of the nanostructured carbide samples, handling was performed under Ar atmosphere.

## 3. Results

**Figure 3:**
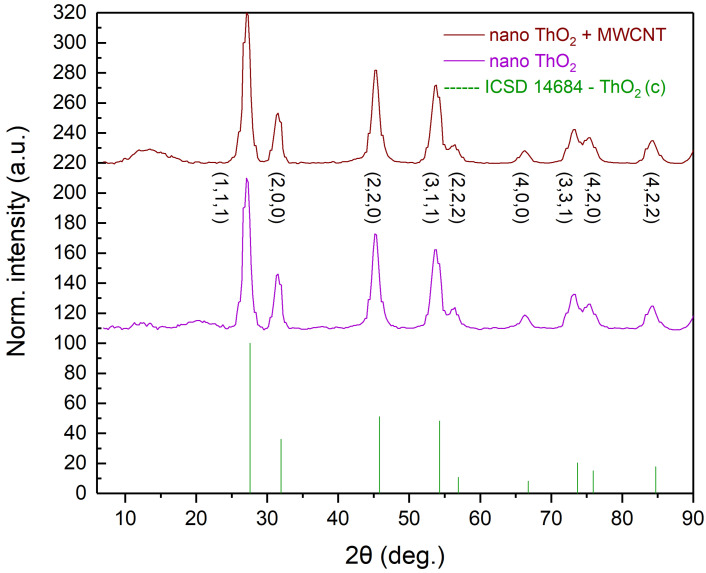
XRD patterns of the ${\mathrm{nano-ThO}}_{2}$ precursor before and after adding MWCNT.

**Figure 4:**
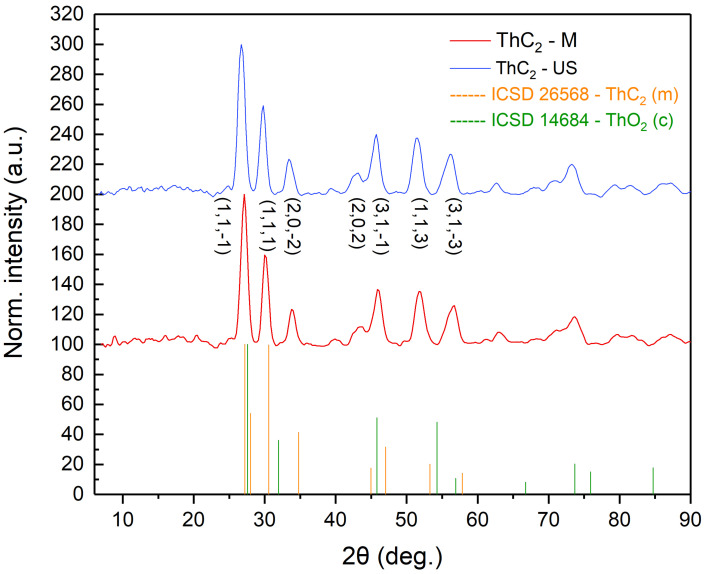
XRD patterns of ${\mathrm{ThC}}_{2\pm x}$ produced via ultrasound bath and via finger probe (ThC-M).

Powder XRD was performed using a Bruker D8 diffractometer with Mo K-alpha source in a Bragg–Brentano geometry. Figure 3 shows the nano ${\mathrm{ThO}}_{2}$ with MWCNT green body samples and Figure 4 shows the two carbothermally reduced samples. The resulting XRD patterns were converted to Cu K-alpha wavelength for easier comparison. The precursor nanostructured thoria exhibits a fluorite structure. The pattern suffered from high background noise, particularly in the 2θ = 6 to 20° and 60 to 90° regions, and was not further refined. It can be observed that the addition of the MWCNT results in the appearance of a wide band in the 2θ = 10 to 15 range stemming from disordered carbon, with this band being absent in both carbothermally reduced thorium carbide samples (Figure 4), along with the characteristic peaks in the thoria precursor, evidencing that the conversion from oxide to carbide was successful. Furthermore, we also observe, in both carbide samples, a peak shift, which could indicate a different stoichometry than ${\mathrm{ThC}}_{2}$ and a distortion of the monoclinic structure. A similar shift in the diffraction peaks was previously reported by Vuong in MWCNT-based uranium di-carbide [13].

**Figure 5:**
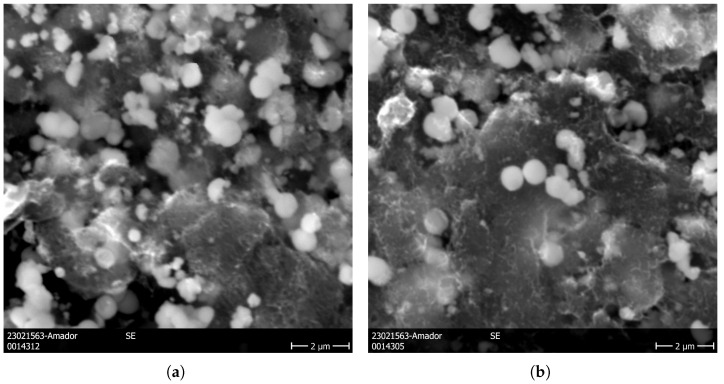

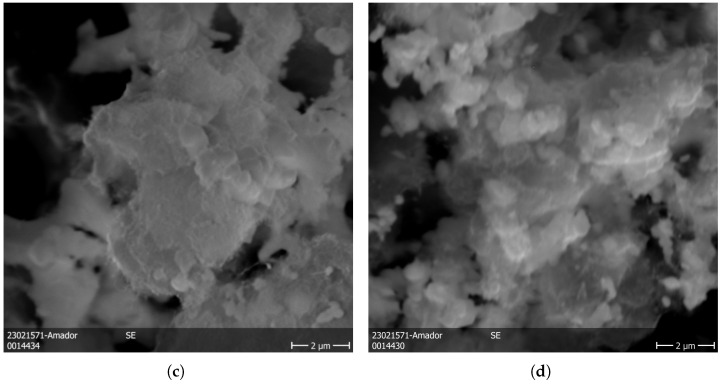
SEM images of ${\mathrm{ThO}}_{2}$-M (**a**) and ${\mathrm{ThO}}_{2}$-US precursors (**b**) and the carbothermally reduced ${\mathrm{ThC}}_{x}$-M (**c**) and ${\mathrm{ThC}}_{x}$-US (**d**).

Samples of the carbide, as well as both precursor mixes (obtained from ultrasound bath and finger probe), were taken to a ThermoFisher Scientific Quattro S SEM equipped with a 30 kV Field Emission Gun, a secondary electron Everhart–Thornley Detector (ETD), and a Bruker EDS XFlash 6/30 silicon drift detector, installed inside an inert-atmosphere glovebox. No clear differences were observed between both methods of mixing (Figure 5). A higher level of resolution would be required to study nanoparticle agglomeration levels. For the carbide, the individual nanoscale clusters of thoria observed in the precursor were not observed in the final product, and the MWCNT structure was harder to spot but is still present (findings further supported by Raman spectroscopy data), fulfilling the goal of the synthesis to obtain a hybrid material with MWCNT bridges through which isotopes may escape thorium carbide and be efficiently extracted from the material. EDS analysis was performed on precursor and carbide samples (see Appendix A Supporting Information) produced with the two preparation methods by rastering a representative area, with Al residues found in both of the precursor samples but absent from the two final product samples. Upon further analysis of the raw materials via SEM-EDS, it became evident that the contamination originated from the batch of MWCNT used in the preparation of both samples.

**Figure 6:**
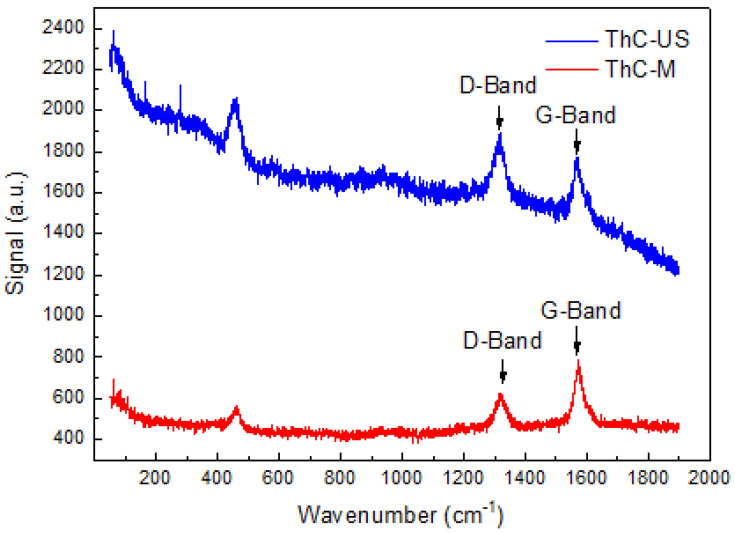
Raman spectra of ThC-M and ThC-US samples, with the MWCNT active D and G bands highlighted.

The differences in the final product obtained using both both precursor production methods can be seen in the Raman spectra (Figure 6), where the D and G bands are close to the natural ratios observed in pristine MWCNT [14] in the sample that was mixed with the ultrasonic finger (ThC-US), whereas in the sample that was sonicated in the water bath, there is a nearly 1:3 ratio between the D and G bands, indicating that the MWCNT structure survived the carbothermal reduction process. In the latter case, a non-quantifiable degree of functionalization of the surface of the MWCNT with hydroxyl or carboxylic groups on the hybridized ${\mathrm{sp}}^{2}$ carbon atoms in the CNT structure occurred, where they may have been modified by interaction with the solvent (isopropyl alcohol) during sonication, an effect that has been widely studied for MWCNT-based dispersions [14,15,16]. The Raman spectra for the precursor samples were dominated by the presence of zinc stearate (which is Raman-active in the relevant wavenumber region) as a pressing aid and no information could be gleaned from them.

## 4. Conclusions

It is demonstrated that there is a viable process to produce nanostructured thorium–carbide in a carbon nanotube scaffold where the thorium precursor is produced using a bottom-up approach. The resulting material shows improvements compared to previous attempts to produce nanostructured thorium carbide [7]. The work can potentially be translated to other carbide materials already employed as ISOL target materials, such as lanthanum or uranium, where similar sintering problems have been observed to occur when operating these materials at high operating temperatures, or even to materials produced with different carbon allotropes. Further testing would be required in the typical temperature regimes used for online operation in ISOL facilities (e.g., by adding a tracer element and studying its release in an offline setup) to ensure the long-term integrity of the nanostructure and its effectiveness in delaying densification at operating temperatures above 2100 °C.

## Figures and Tables

**Figure A1:**
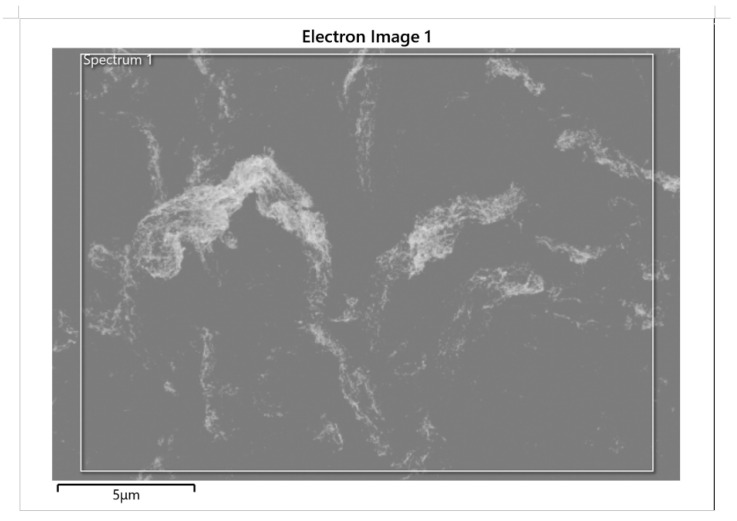
SEM image of pristine MWCNT.

**Figure A2:**
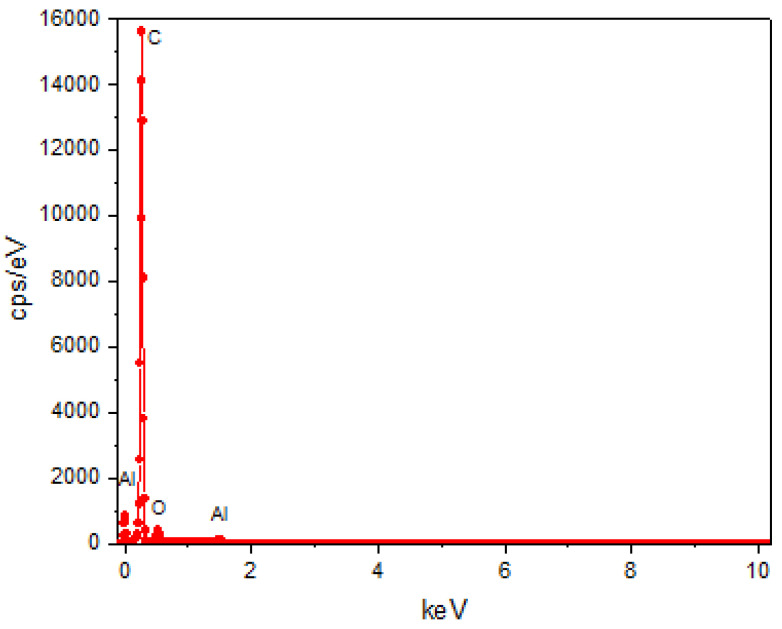
EDS spectrum of pristine MWCNT.

**Figure A3:**
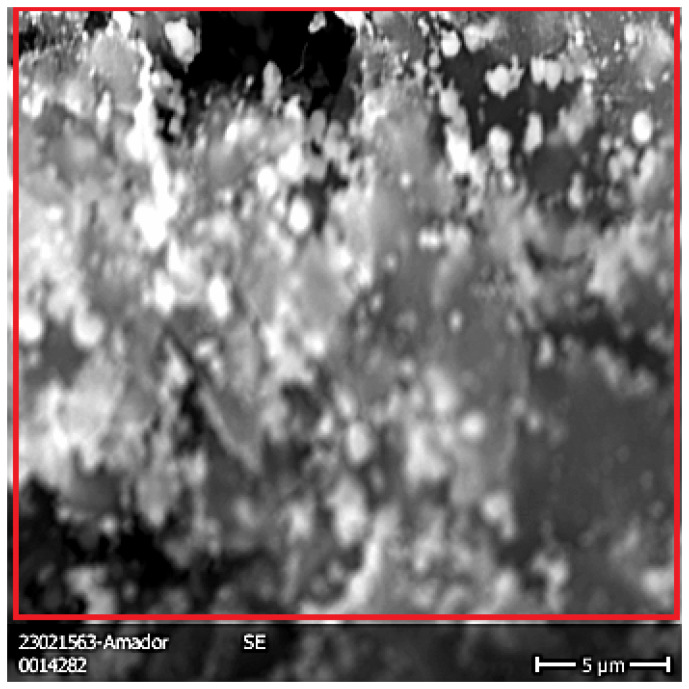
SEM image of ${\mathrm{ThO}}_{2}$ + MWCNT prepared via ultrasound bath (${\mathrm{ThO}}_{2}$-M).

**Figure A4:**
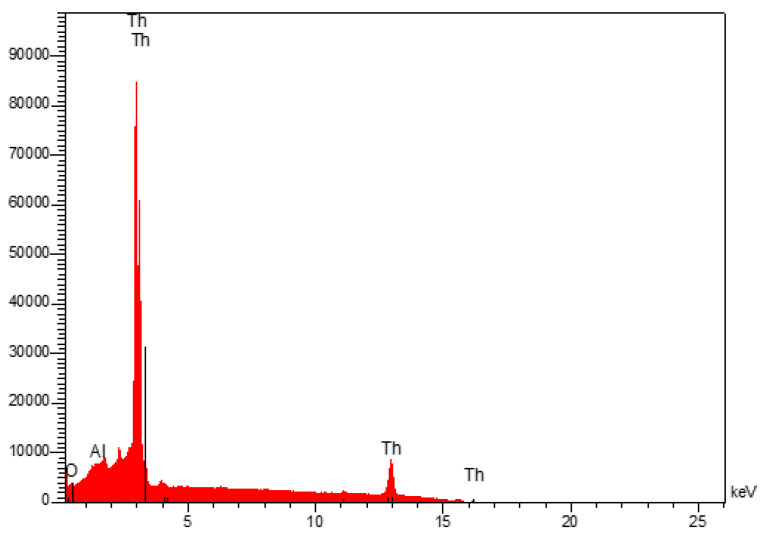
EDS spectrum of ${\mathrm{ThO}}_{2}$ + MWCNT prepared via ultrasound bath (${\mathrm{ThO}}_{2}$-M).

**Figure A5:**
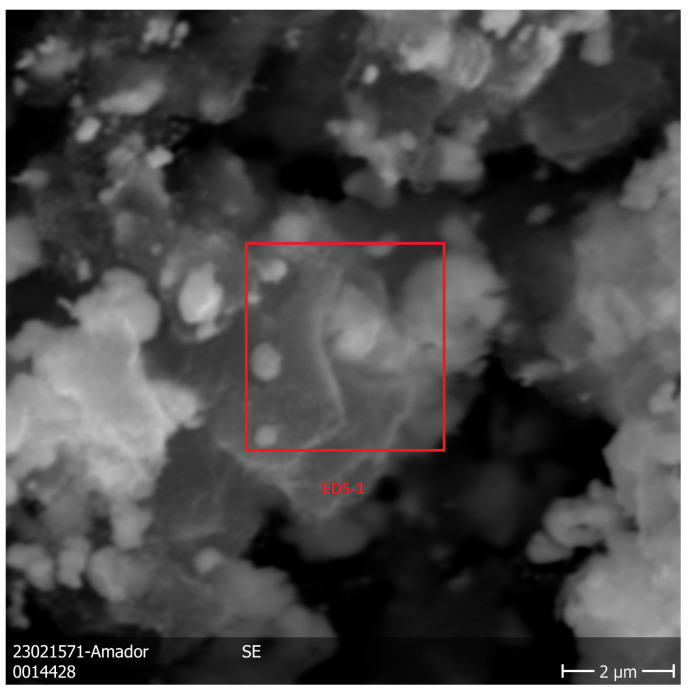
SEM image of ${\mathrm{ThO}}_{2}$ + MWCNT prepared via ultrasound bath (${\mathrm{ThO}}_{2}$-M).

**Figure A6:**
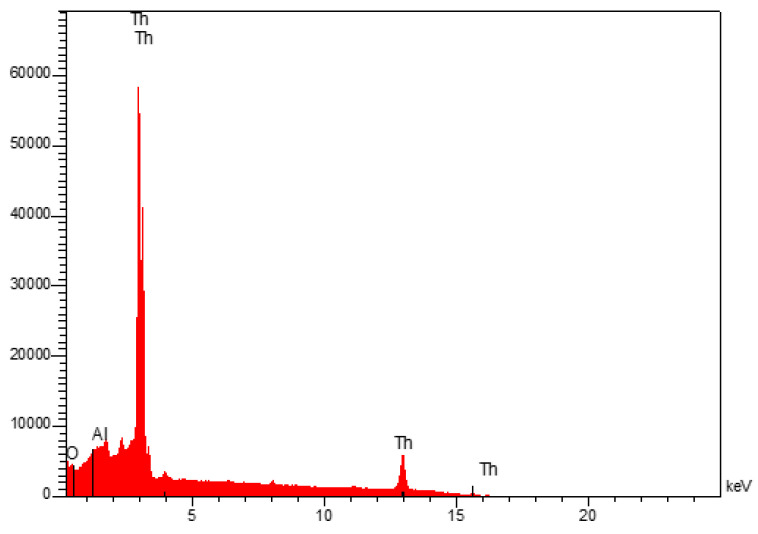
EDS spectrum of ${\mathrm{ThO}}_{2}$ + MWCNT prepared via ultrasound bath (${\mathrm{ThO}}_{2}$-M).

**Figure A7:**
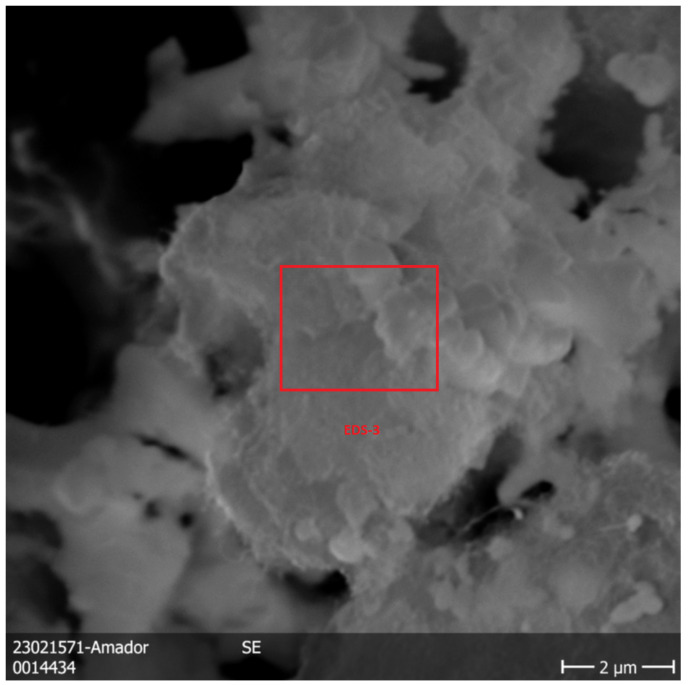
SEM image of thorium carbide (ThC-M).

**Figure A8:**
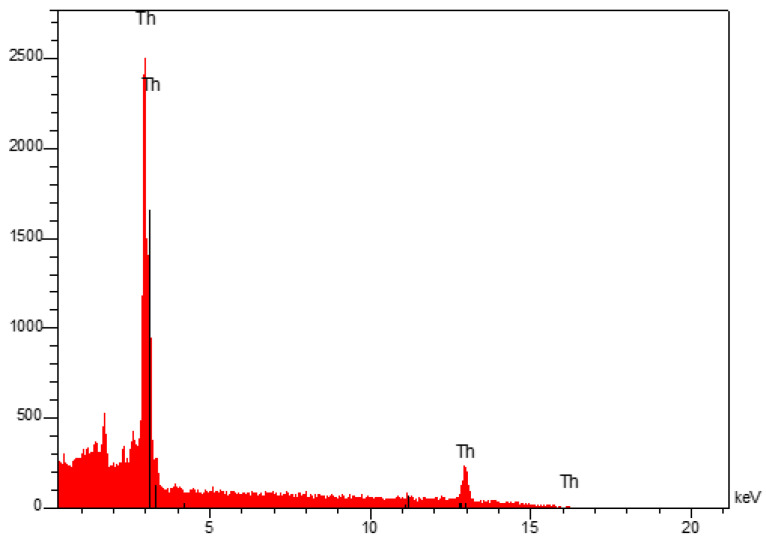
EDS spectrum of thorium carbide (ThC-M).

**Figure A9:**
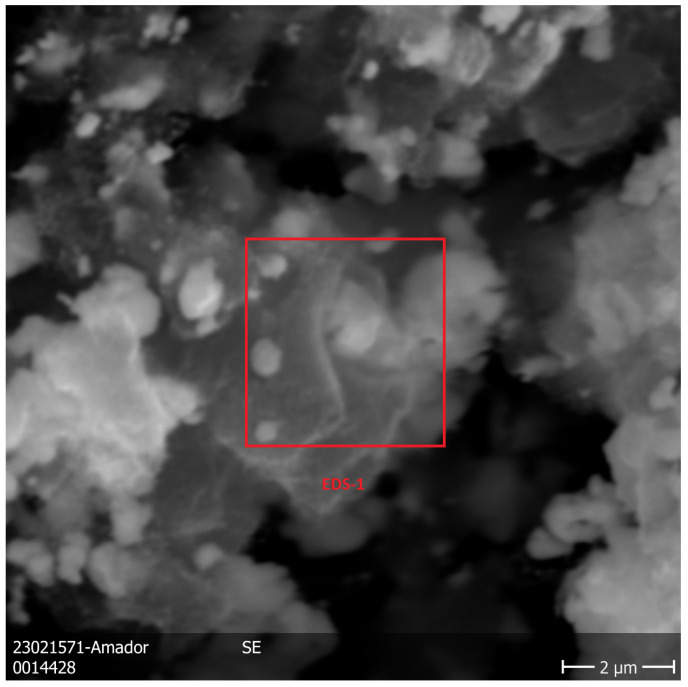
SEM image of thorium carbide (ThC-US).

**Figure A10:**
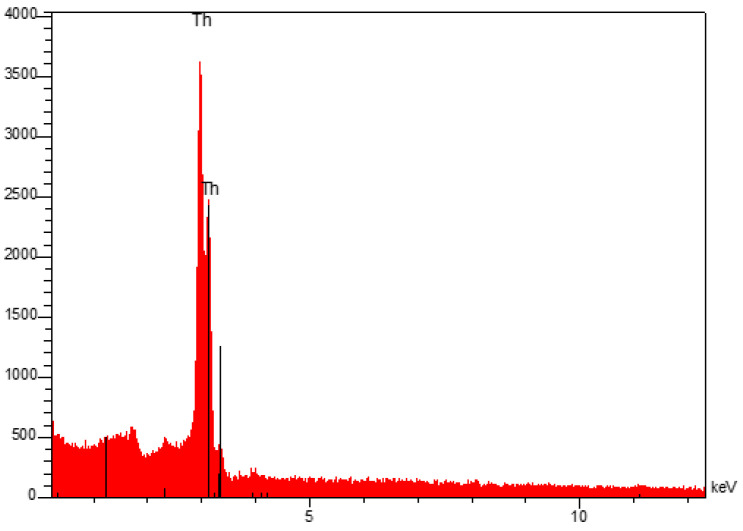
EDS spectrum of thorium carbide (ThC-US).

## Data Availability

Data are contained within the article.

## Abbreviations

The following abbreviations are used in this manuscript:

CERN	The European Organization for Nuclear Research
ISOLDE	Isotope Separation On-Line DEvice
MWCNT	Multi-Walled Carbon NanoTubes
ISOL	Isotope Separation On-Line
CNT	Carbon NanoTubes
NP	Nanoparticle
SEM	Scanning Electron Microscopy
EDS	Energy-Dispersive Spectroscopy
PXRD	Powder X-Ray Diffraction
XRD	X-ray Diffraction
SiC	Silicon Carbide
CaO	Calcium Oxide
CO	Carbon Monoxide
UC	Uranium Carbide
ThC	Thorium Carbide

## Appendix A. Supporting Information

EDS spectra were taken from the carbon nanotubes (Figure A1 and Figure A2 and Table A1), from the precursor sample (${\mathrm{ThO}}_{2}$-M) via ultrasound bath dispersion (Figure A3 and Figure A4 and Table A2), from the precursor sample (${\mathrm{ThO}}_{2}$-US) via ultrasonic finger treatment (Figure A5 and Figure A6 and Table A3), and from the two resulting carbides (for ThC-M in Figure A7 and Figure A8 and ThC-US in Figure A9 and Figure A10). Tables are not presented for the carbide samples as only thorium could be confidently identified due to carbon being a low-Z element (and as the carbon ratio decreased from the six equivalents of thorium present in the precursor mixture to around two in the final product. The MWCNT EDS and corresponding SEM images were obtained posteriorly in a Zeiss FIB-SEM XB40 equipped with an Oxford Instruments EDS apparatus.

**Table A1:** EDS results for MWCNT, Nanocyl NC7000.

Element	Weight%	Atomic%
C	94.1	96.7
O	3.0	2.3
Al	2.1	0.95

**Table A2:** EDS results for ${\mathrm{ThO}}_{2}$ + MWCNT prepared via ultrasound bath (${\mathrm{ThO}}_{2}$-M).

Element	Weight %	Atomic %
Th	93.4	49.0
O	4.0	30.2
Al	1.0	4.6

**Table A3:** EDS results for ${\mathrm{ThO}}_{2}$ + MWCNT prepared via ultrasound bath (${\mathrm{ThO}}_{2}$-M).

Element	Weight%	Atomic%
Th	84.3	29.6
O	8.4	42.5
Al	5.9	17.7
